# Percutaneous removal of a knotted Swan–Ganz catheter

**DOI:** 10.1007/s12928-021-00822-2

**Published:** 2021-11-08

**Authors:** Hibiki Mima, Soichiro Enomoto, Yodo Tamaki, Makoto Miyake, Hirokazu Kondo, Toshihiro Tamura

**Affiliations:** Department of Cardiology, Tenri Hospital, 200 Mishima-cho, Tenri, Nara 632-8552 Japan

A 48-year-old man who had undergone aortic valve surgery 1 week previously became hemodynamically unstable in the intensive care unit. His physician decided to insert a Swan–Ganz catheter (SGC) (Edwards Lifesciences, Tokyo, Japan) without fluoroscopic guidance. The SGC was inserted via the right internal jugular vein and advanced into the right ventricle, but it could not be advanced into the pulmonary artery. After repeated attempts, the physician pulled back the SGC, but it could not be retrieved. X-ray examination showed knot formation in the SGC, and the patient was referred to us.

First, a new 5-Fr sheath was placed in the right internal jugular vein, and a 0.014-inch guidewire (Cruise; Asahi Intecc, Tokyo, Japan) was passed through the center of the knot. A 6-mm balloon (Rx-Genity; Kaneka, Tokyo, Japan) was inflated to expand the knot diameter; however, the knot was not completely untied. A 10-mm-diameter Amplatz Goose Neck snare (Medtronic, Dublin, Ireland) was then advanced to the distal end of the SGC along with the Cruise wire. Finally, we grasped the tip of the SGC and pulled it back, causing untying of the knot and successful removal of the SGC.

**Fig. 1:**
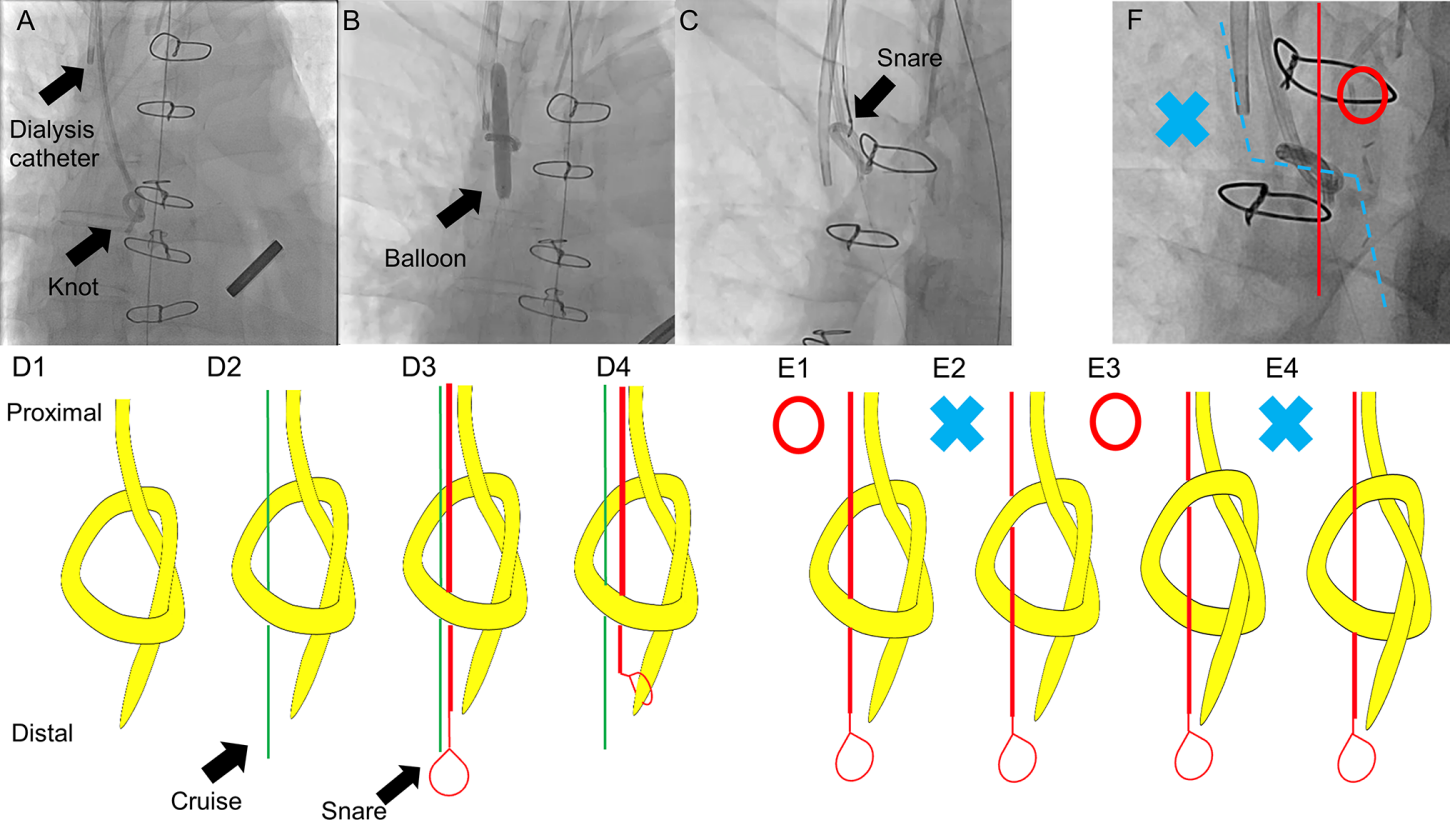
**a** Knot formation in the Swan–Ganz catheter. **b** Balloon dilatation loosened the knot. **c** The tip was pulled back by the snare passing through the knot. **d**1–4 Schematic diagram of the procedure. **e**1–4 The snare must be on the same side as the tip of the Swan–Ganz catheter. **f** Rotational angiography showed whether the snare was on the correct path (red line) or not (blue line). Fortunately, the knot was tilted, which enabled the snare to follow the correct path easily

Several interventional radiological strategies to remove knotted catheters have been reported, including dilating the knot by large-diameter balloons [1], covering the knot with a larger sheath, and pulling the catheter end back through the knot following balloon dilatation [2], which is the basis of our procedure and might be the most promising. However, this method has a possibility of creating another knot by passing the snare from the wrong side as shown in Fig. 1e2 and 4. Rotational angiography is required to check whether the snare is on the same side as the tip of the SGC, although the knot is likely to tilt to a preferred angle for the correct path (Fig. 1f). Sufficient knot dilation by adequately sized balloons is also important to avoid migrating the whole knotted catheter without untying. Surgical removal is required in some situations. If the knot is very large or multiple knots exist, pulling the catheter can cause vascular trauma. Furthermore, intracardiac structures may be injured if the knot is entrapped in the chordae tendineae or heart valves [3].

In conclusion, physicians should be aware of this rare complication and manipulate catheters carefully during blind procedures.
